# Optimizing Infusion Center Efficiency in Multiple Sclerosis: A Nursing Multicenter Survey on Organizational Impact of Ublituximab

**DOI:** 10.3390/healthcare14142094

**Published:** 2026-07-13

**Authors:** Martina Campobasso, Elena Mutta, Caterina Giovanna Moscato, Salvatorica Carta, Roberta Lorenzi, Elisa Bressan, Enzo Troncone, Francesco Pastore

**Affiliations:** 1 UOSD Multiple Sclerosis Centre, Policlinic Tor Vergata, 00133 Rome, Italy; martina.campobasso@gmail.com; 2 SISM Italian Society of MS Nurses, 16149 Genova, Italy; 3 Neurology Unit with a Neuroimmunology Focus, ASST Valle Olona, 21013 Gallarate, Italy; elena.mutta@asst-valleolona.it; 4 Neurology Unit, Sant’Andrea Hospital, 00189 Rome, Italy; caterac@libero.it; 5 SC Multiple Sclerosis Center, Binaghi Hospital, 09126 Cagliari, Italy; salvatorica.carta@aslcagliari.it; 6 Neurology Unit (UOSD), Muscatello Hospital, 96011 Augusta, Italy; roberta.lorenzi@asp.sr.it; 7 AULSS 8 Berica—Multiple Sclerosis Center, 36100 Vicenza, Italy; elisa.bressan@aulss8.veneto.it; 8 Butterfly Decisions, 84122 Salerno, Italy; enzo@butterflydecisions.com; 9 Department of Precision and Regenerative Medicine and Ionian Area (DiMePRe-J), University of Bari Aldo Moro, 70121 Bari, Italy

**Keywords:** multiple sclerosis, infusion therapy, infusion centers, nursing workload, healthcare organization, workflow efficiency, monoclonal antibodies, patient-centered care, ublituximab, health services research

## Abstract

**Highlights:**

**What are the main findings?**

- Infusion capacity in multiple sclerosis centers is primarily driven by organizational factors (resource availability and workload), rather than by the duration of nursing care activities.
- Significant inefficiencies persist in infusion workflows, with chair occupancy and pathway organization emerging as key system bottlenecks beyond pharmacological infusion time.

**What are the implications of the main findings?**

- Shorter infusion therapies (e.g., one-hour protocols such as ublituximab) may act as enablers of organizational redesign, improving patient flow and center efficiency rather than directly reducing workload.
- Optimizing infusion pathways requires restructuring care processes to preserve relational time and enhance patient-centered care, alongside improving operational efficiency.

**Abstract:**

**Background:** The increasing use of infusion therapies for multiple sclerosis (MS) has improved disease management but has also increased organizational complexity and nursing workload within infusion centers. Evidence on the organizational implications of infusion care and the perspectives of MS nurses remains limited. This study aimed to characterize the organization of Italian MS infusion centers, explore nursing workload and operational challenges, and assess nurses’ perceptions regarding the potential organizational implications of shorter infusion protocols. **Methods:** A sequential two-phase study was conducted. First, a multicenter cross-sectional survey was administered to nurses working in Italian MS infusion centers (July–September 2025). Subsequently, a national expert board was convened (March 2026) to discuss and contextualize the survey findings. Descriptive statistics, multivariable linear regression, and non-parametric analyses were performed. **Results:** Sixty-eight MS nurses participated (response rate: 87.2%). Infusion centers were characterized by high patient volumes, limited staffing, frequent multitasking, and substantial administrative burden. Post-infusion monitoring represented the most time-consuming phase of care. Resource availability was independently associated with the number of daily infusions (β = 0.46, *p* < 0.001), whereas time devoted to nursing assistance and operational workload were not. Expert panel discussions highlighted chair occupancy, workflow organization, and the distinction between technical and relational nursing time as key organizational issues. Nurses perceived shorter infusion protocols as a potential opportunity to improve scheduling flexibility and patient flow, while emphasizing that any organizational benefits would depend on broader workflow redesign. **Conclusions:** Organizational capacity in MS infusion centers appears to be driven primarily by structural resources rather than nursing task duration. Nurses identified several opportunities to improve infusion pathways and suggested that shorter infusion protocols may support organizational optimization. However, these potential advantages should be regarded as hypotheses based on nurses’ perceptions and require confirmation in prospective comparative studies.

## 1. Introduction

### 1.1. Organizational Challenges in Infusion Centers

The management of infusion therapies in patients with multiple sclerosis (MS) represents a critical organizational and clinical challenge for healthcare systems [1]. The increasing use of monoclonal antibodies has significantly improved disease control and patient outcomes [2]. However, it has also placed substantial pressure on infusion centers in terms of resource allocation, workflow organization, and nursing workload [3]. The increasing demand for infusion therapies has also raised concerns regarding the long-term sustainability of infusion center services. Rising patient volumes, limited physical capacity, and shortages of specialized nursing personnel may compromise both efficiency and quality of care, highlighting the need for innovative organizational models capable of optimizing available resources while maintaining high standards of patient-centered care. Infusion therapies for MS, particularly those requiring prolonged administration times, are associated with extended chair occupancy, increased staff engagement, and reduced turnover capacity within infusion units [4]. To improve infusion center efficiency and ensure sustainable care delivery, it is essential to understand current organizational models, resource allocation, and barriers affecting clinical workflows.

### 1.2. Impact of Infusion Time on Patient Experience

At the same time, prolonged infusion stays may transform infusion settings into meaningful relational spaces, where the care environment and interactions with healthcare professionals and peers can support information exchange, emotional support, and patient engagement [5]. However, both excessively prolonged and markedly reduced infusion times may negatively impact patients’ quality of life: on the one hand, extended stays (e.g., intravenous ocrelizumab, alemtuzumab) can be physically and psychologically burdensome [6]; on the other hand, very short administration times (e.g., subcutaneous ocrelizumab, natalizumab) may limit opportunities for interaction, support, and information exchange within the care setting. Consistently, real-world data on intravenous monoclonal antibody administration highlight a substantial time burden, with patients spending several hours per visit in infusion centers and frequently reporting stress related to these prolonged treatments, which may also negatively affect the quality of communication with healthcare professionals, making interactions more demanding and less effective [7].

Furthermore, evidence from oncology settings suggests that, although subcutaneous formulations can reduce administration time, the overall time spent in clinical settings often remains only marginally improved due to workflow inefficiencies and non-treatment-related activities, ultimately limiting both the perceived benefit for patients and the quality of communication within the care process [8]. In the specific setting of multiple sclerosis, a large proportion of patients receiving infusion therapies report that these moments represent an opportunity to interact with healthcare professionals (74.4%) and to share experiences with other patients (61.1%) [9]. However, this potential may be compromised in the absence of well-organized infusion services, particularly when infusion times are either excessively prolonged or overly shortened, limiting the quality and effectiveness of these interactions.

### 1.3. Role of Nursing

Among all healthcare professionals involved in the patient journey, MS nurses are the most directly engaged in the drug administration process and are therefore best positioned to capitalize on care time, both in terms of clinical management and the therapeutic relationship [10,11]. Within this context, nurses play a pivotal role, being directly responsible for patient assessment prior to infusion, preparation and administration of therapies, continuous monitoring for infusion-related reactions, and patient education [12]. Beyond their technical responsibilities, MS nurses play a key role in promoting treatment adherence, health literacy, self-management behaviors, and patient empowerment. Consequently, organizational changes affecting infusion delivery may have important implications for nursing-sensitive outcomes, including patient satisfaction, perceived quality of care, therapeutic alliance, and engagement in disease management. Importantly, infusion sessions are not merely technical procedures but represent critical moments for comprehensive nursing evaluation, including the assessment of clinical stability, treatment adherence, symptom progression, and patient-reported outcomes [10]. Optimised infusion duration has the potential not only to improve patient journey and resource utilization but also to alleviate nursing workload and free up time for more comprehensive clinical assessment and patient interaction [13]. At the same time, such optimization may help rebalance the dual function of infusion settings as both treatment and relational environments [14]. This shift could enhance the quality of care, allowing nurses to focus more on clinical decision support, early detection of adverse events, and reinforcement of self-management strategies [15].

### 1.4. Opportunities with Faster Therapies

In this context, the optimization of infusion centre efficiency has become a priority, especially considering emerging therapies characterized by shorter infusion times [16]. Monoclonal antibody with faster infusion may represent a valuable organizational alternative, as it can be safely infused in as little as 1 h, potentially balancing time efficiency with the preservation of meaningful nurse–patient interactions and patient-cantered care.

### 1.5. Knowledge Gaps and Study Rationale

Despite the growing availability of infusion therapies with different administration times, evidence remains limited regarding how multiple sclerosis infusion centers are organized in routine practice, how nursing workload is distributed, and which operational barriers may hinder the optimization of care delivery [17]. While most studies evaluating infusion therapies have focused on efficacy, safety, and patient satisfaction, considerably less attention has been devoted to the organizational implications of infusion duration and workflow management. Moreover, the perspectives of MS nurses, who coordinate much of the infusion process and spend the greatest amount of direct time with patients, remain largely underrepresented in the literature. Nurses, being the healthcare professionals most directly involved in infusion management, are uniquely positioned to identify inefficiencies, unmet needs, and opportunities for improvement across the patient journey [18]. To date, little is known about how infusion duration affects nursing workload, resource utilization, and patient-centered care in real-world MS infusion centers. Addressing these gaps is essential to inform evidence-based strategies for optimizing infusion services. In this perspective, a structured multicenter survey may provide a real-world overview of current infusion practices, organizational constraints, and perceived priorities for change, generating the evidence needed to inform the development of a more efficient and patient-centered model of infusion care. Therefore, the aim of this study was to investigate the organization of MS infusion centers in Italy from the nurses’ perspective, assess the perceived impact of infusion duration on clinical practice, and explore the potential organizational implications of therapies with shorter administration times, such as ublituximab. Understanding nurses’ perceptions is particularly relevant because they are often the first professionals to identify workflow inefficiencies, unmet patient needs, communication barriers, and opportunities for service redesign. Their experience can therefore provide unique insights into how emerging therapies may influence both operational efficiency and the overall patient experience.

## 2. Materials and Methods

### 2.1. Study Design

This study adopted a sequential two-phase design. The first phase consisted of a multicenter cross-sectional survey conducted between July and September 2025 among nurses working in MS infusion centers across Italy. The second phase consisted of a national online expert board held in March 2026, aimed at discussing, contextualizing, and validating the survey findings and identifying their implications for clinical practice, nursing workload, and infusion center organization.

### 2.2. Participants

Participants were recruited through the Italian Multiple Sclerosis Nursing Network. Eligible participants were nurses actively involved in infusion therapy management and in the clinical care of people with MS. Participation was voluntary. All participants received information about the aims of the study before completing the questionnaire and provided informed consent prior to participation.

### 2.3. Data Collection

Survey data were collected electronically using the Butterfly Decisions AI-Assisted Decision-Making platform (https://www.butterflydecisions.com), in compliance with the General Data Protection Regulation (GDPR, Regulation EU 2016/679). The survey was distributed to eligible nurses through the Italian Multiple Sclerosis Nursing Network. The platform allowed anonymous and secure data entry. Responses were stored in a centralized database and exported for analysis. AI-assisted functionalities embedded within the platform were used only to support the organization and thematic summarization of qualitative comments and expert board discussions. AI outputs were not used to generate statistical results or study conclusions. All AI-generated summaries were independently reviewed and validated by the Scientific Committee before inclusion in the analysis [19,20].

### 2.4. Questionnaire Development

A structured ad hoc questionnaire was developed to explore infusion center organization, nursing workload, resource availability, infusion workflows, and the perceived impact of infusion duration in MS care. Questionnaire development was informed by a review of the available literature on infusion therapy workflows, nursing workload, resource allocation, and chronic disease care organization. The initial version was refined through consultation with nurses experienced in MS infusion services to ensure relevance to real-world clinical practice. Face and content validity were assessed by a panel of MS nurses with expertise in infusion management and clinical care. Feedback from the panel was used to refine item wording, clarity, and relevance. A pilot assessment was subsequently conducted among a small group of nurses to evaluate comprehensibility, feasibility, and completion time before national dissemination. The final questionnaire included 60 items covering operator characteristics, organizational features of infusion centers, resource availability, operational processes, infusion protocols, and key performance indicators. Response formats included numeric variables, categorical responses, Likert-type scales, and open-ended questions. The complete item list is reported in Supplementary File S1. For the purpose of the present analysis, selected variables were grouped a priori into three composite domains. Time of Nursing Assistance represented the mean time (minutes) spent across the main phases of infusion care, including preparation of the infusion setting, patient reception and assessment, clinical setup, post-infusion monitoring, discharge management, patient education, and caregiver communication. Operational Workload reflected nursing workload intensity and was calculated as the arithmetic mean of the average number of patients manageable per nurse per shift and the number of patients managed simultaneously during the infusion shift. Resource Availability reflected the structural and staffing capacity of infusion centers and was calculated as the arithmetic means of the number of active infusion stations, infusion pumps, and nurses available per infusion shift. These composite domains were defined as a priori to summarize conceptually related organizational indicators into clinically meaningful constructs and to reduce dimensionality in the statistical analyses. The composite domains were developed specifically for this study as exploratory organizational indicators and were not intended as psychometrically validated measurement scales (Appendix A).

### 2.5. Expert Board

A national online expert board was conducted in March 2026 to discuss the main findings emerging from the survey. The board included seven healthcare professionals with expertise in MS care, infusion management, and nursing leadership. Experts were purposively selected to ensure representation from different geographical areas and clinical settings across Italy. The discussion focused on the interpretation of survey results, the identification of organizational challenges, and the definition of priority areas for improvement in MS infusion centers. The Butterfly Decisions platform supported the organization of the discussion transcript and thematic summarization. These outputs were reviewed and validated by the Scientific Committee before being used to support the interpretation of findings [19,20].

### 2.6. Data Analysis

Descriptive statistics were used to summarize sample and organizational characteristics. Continuous variables were reported as mean and standard deviation or median and range, according to distribution. Categorical variables were expressed as frequencies and percentages. The distribution of continuous variables was assessed using the Shapiro–Wilk test. Given the non-normal distribution of several variables, non-parametric tests were applied when appropriate. To investigate factors associated with infusion activity, a multivariable linear regression model was performed using the number of infusions supervised per day as the dependent variable. Independent variables included the three composite domains—Time of Nursing Assistance, Operational Workload, and Resource Availability—together with selected organizational variables, including monthly patient volume and perceived impact of long infusion times on nursing workload. Composite domain scores were calculated by standardizing the contributing variables and combining them into summary scores. Higher scores indicated higher levels of the corresponding construct. Model fit was assessed using R^2^ and adjusted R^2^. Regression coefficients were reported with standard errors, standardized estimates, and 95% confidence intervals.

Differences across groups were evaluated using the Kruskal–Wallis test, with effect size reported as epsilon squared (ε^2^). When significant, post hoc pairwise comparisons were performed using the Dwass–Steel–Critchlow–Fligner test to account for multiple comparisons. All statistical analyses were performed using jamovi version 2.6. Statistical significance was set at *p* < 0.05. AI-assisted analysis was limited to the organization and thematic summarization of qualitative expert board discussions. AI was not used for inferential statistical analyses, formal qualitative coding, or generation of study conclusions. All AI-assisted outputs were checked against the original discussion content by the research team [19,20].

### 2.7. Ethical Considerations

The study was conducted in accordance with the principles of the Declaration of Helsinki and applicable data protection regulations, including the General Data Protection Regulation (GDPR, Regulation EU 2016/679). Participation was voluntary, and informed consent was obtained before survey completion. Completion and submission of the online questionnaire were considered indicative of informed consent. Data were collected anonymously, and no personally identifiable information was recorded. According to current Italian regulations, ethics committee approval was not required because the study consisted of an anonymous, voluntary, non-interventional online survey conducted among healthcare professionals, with no involvement of patients and no collection of sensitive personal or clinical data. This approach is consistent with the provisions outlined by the Italian Medicines Agency (AIFA) Determination No. 425 of 2024 regarding observational and non-interventional studies.

## 3. Results

### 3.1. Sample Characteristics

A total of 68 nurses from multiple sclerosis centers across Italy completed the survey (response rate 87.2%). Participants reported extensive professional experience, with a mean of 21.54 years as nurses (SD = 10.52) and 8.66 years of experience specifically in multiple sclerosis care (SD = 7.97). The average number of infusions supervised per day was 7.79 (SD = 5.36), indicating substantial daily clinical activity (Table 1).

Regarding organizational structure, centers treated on average 95.82 patients per month receiving infusion therapies (SD = 87.81), highlighting marked variability across settings. The mean number of infusion chairs was 5.96 (SD = 3.42), with 7.06 operational infusion stations (SD = 7.24) and 5.87 infusion devices (SD = 4.78). The average number of nurses per infusion shift was 2.44 (SD = 1.39). In terms of patient management, nurses reported managing 5.98 patients per shift on average (SD = 4.91) and 2.54 patients simultaneously (SD = 3.73). Most centers reported the use of infusion pumps (86.8%), while 11.8% reported partial use and 1.5% no use. Access to standardized protocols was almost universal (97.1%). A dedicated infusion area was available in 57.4% of centers, while 42.6% operated in shared settings. Monthly patient volume varied, with 38.2% of centers treating more than 50 patients, 27.9% between 30 and 50, 27.9% between 10 and 30, and 5.9% fewer than 10 patients. Nursing shifts were predominantly fixed (75.0%), with 14.7% of centers adopting flexible schedules and 10.3% variable shifts depending on workload. Administrative or bureaucratic activities between infusions were reported by 88.2% of respondents. In terms of workload and operational demand, nurses reported working an average of 2.49 additional hours per week beyond scheduled time due to infusion-related activities (SD = 3.69). The perceived workload during infusions had a mean score of 3.84 (SD = 0.77) on a 5-point scale, indicating a generally moderate-to-high workload intensity. The infusion process required a considerable amount of time across all phases (Table 2 and Table 3).

Preparation of the infusion setting required 13.90 min on average (SD = 6.87), patient reception and assessment 16.31 min (SD = 9.52), and vital signs assessment with premedication and infusion setup 27.74 min (SD = 21.15). Post-infusion monitoring was the most time-consuming phase, with a mean duration of 52.50 min (SD = 21.54), followed by post-infusion management activities such as discharge preparation (17.37 min, SD = 12.30). Time dedicated to patient education and relationships averaged 20.66 min (SD = 13.71), while communication with caregivers required 14.03 min (SD = 8.36). Bureaucratic activities required an additional 18.07 min on average (SD = 17.19). Operational inefficiencies were also highlighted, with an average waiting time of 20.85 min from patient arrival to infusion start (SD = 14.61) and a mean chair idle time of 19.03 min between infusions (SD = 22.38) (Table 4).

Regarding perceived impact, 51.5% of nurses reported that prolonged infusion times had a moderate impact on workload, 25.0% reported a high impact, 13.2% a low impact, and 10.3% a no impact. Delays in access to infusion therapies were commonly reported: 52.9% indicated occasional delays and 16.2% frequent delays for first infusions, while 30.9% reported no delays. For subsequent infusions, 66.2% reported no delays, 32.4% occasional delays, and 1.5% frequent delays. On the scheduled infusion day, 51.5% reported no delays, 42.6% occasional delays, and 5.9% frequent delays (Table 5).

Finally, composite domains were calculated to summarize key aspects of infusion care. The mean Time of Nursing Assistance was 22.11 min (SD = 6.91). The Operational Workload index, representing nursing workload intensity, had a mean value of 5.71 (SD = 4.23), while the Resource Availability index, representing structural capacity, had a mean of 5.15 (SD = 3.14), reflecting variability in both workload intensity and structural capacity across centers (Table 6).

### 3.2. Determinants of Infusion Capacity: Multivariable and Non-Parametric Analyses

#### 3.2.1. Multiple Linear Regression Analysis

A multiple linear regression analysis was conducted to identify factors associated with the number of infusions administered per day (N = 68). The overall model was statistically significant and explained 46.7% of the variance in the dependent variable (R = 0.683, R^2^ = 0.467, adjusted R^2^ = 0.384). Among the continuous predictors, resource availability emerged as a significant positive predictor of the number of daily infusions (B = 0.79, SE = 0.18, β = 0.46, t = 4.41, *p* < 0.001), indicating that higher perceived availability of resources was associated with a greater number of infusions performed each day. In contrast, neither time of nursing assistance (B = 0.03, SE = 0.08, β = 0.04, t = 0.39, *p* = 0.697) nor operational workload (B = 0.01, SE = 0.13, β = 0.00, t = 0.04, *p* = 0.965) showed significant associations with the outcome. Regarding monthly patient volume, centers managing 10–30 patients per month reported significantly fewer infusions per day compared with centers managing 30–50 patients per month (B = −3.54, SE = 1.41, β = −0.66, t = −2.51, *p* = 0.015). Similarly, centers managing fewer than 10 patients per month also reported significantly fewer daily infusions (B = −5.33, SE = 2.38, β = −0.99, t = −2.24, *p* = 0.029). No significant difference was observed between centers managing more than 50 patients per month and the reference category (B = −0.51, SE = 1.33, β = −0.09, t = −0.38, *p* = 0.704). With respect to the perceived impact of long infusion times on nursing burden, respondents reporting low impact had significantly fewer infusions per day compared with those reporting high impact (B = −3.91, SE = 1.80, β = −0.73, t = −2.17, *p* = 0.034). The comparisons between moderate impact and high impact (B = −1.60, SE = 1.29, β = −0.30, t = −1.24, *p* = 0.218), as well as between no impact and high impact (B = −3.04, SE = 1.91, β = −0.57, t = −1.59, *p* = 0.117), were not statistically significant (Table 7).

A Kruskal–Wallis test was conducted to examine differences in perceived time of nursing assistance, operational workload, and resource availability according to monthly patient volume categories. No significant differences were observed for time of nursing assistance (χ^2^ = 3.61, df = 3, *p* = 0.306, ε^2^ = 0.054). In contrast, significant differences emerged for both operational workload (χ^2^ = 11.55, df = 3, *p* = 0.009, ε^2^ = 0.172) and resource availability (χ^2^ = 11.20, df = 3, *p* = 0.011, ε^2^ = 0.167), indicating moderate effect sizes. Post hoc pairwise comparisons revealed that for operational workload, centers managing fewer than 10 patients per month differed significantly from centers managing 30–50 patients per month (*p* = 0.033) and from those managing more than 50 patients per month (*p* = 0.019). No other pairwise comparisons reached statistical significance. Regarding resource availability, centers managing 10–30 patients per month differed significantly from centers managing 30–50 patients per month (*p* = 0.024) and from centers managing more than 50 patients per month (*p* = 0.020). No significant differences were found among the remaining comparisons (Table 8).

Differences in perceived time of nursing assistance, operational workload, and resource availability were examined according to whether respondents perceived long infusion times as having an impact on nursing burden. A statistically significant difference was found for time of nursing assistance (Mann–Whitney U = 399, *p* = 0.038). Participants who reported no impact of long infusion times on nursing burden showed higher time of nursing assistance scores (mean = 24.34, SD = 7.36; median = 22.0) compared with those who perceived an impact (mean = 20.46, SD = 6.12; median = 20.0). No significant differences were observed for operational workload (U = 556, *p* = 0.910) or resource availability (U = 534, *p* = 0.697). Mean operational workload scores were similar between respondents reporting no impact (mean = 5.59, SD = 3.65) and those reporting an impact (mean = 5.79, SD = 4.65). Likewise, resource availability scores did not differ significantly between groups (mean = 5.48, SD = 4.17 vs. mean = 4.90, SD = 2.10, respectively). These findings suggest that the perception of long infusion times as burdensome is associated with differences in the amount of nursing assistance time available, whereas perceived workload and resource availability appear comparable between groups (Table 9).

#### 3.2.2. Observations from the Expert Nursing Board

The qualitative component of the project, integrating pre-work responses and Advisory Board discussion, provided an interpretive layer that complemented and expanded the quantitative results. Overall, participants confirmed the relevance of the survey findings and described infusion centers as complex care environments in which organizational constraints, nursing expertise, and treatment innovation continuously interact. The discussion did not simply validate the descriptive data, but helped clarify the mechanisms through which infusion duration, workflow design, and resource limitations influence both system efficiency and quality of care. Four main expert considerations emerged from the expert panel discussion.

#### 3.2.3. Experienced Professionals Operating in Fragile Systems

A first recurring construct concerned the relationship between the high level of professional expertise available in MS centers and the complexity of organizational processes. Participants described nurses as highly experienced professionals, capable of recognizing subtle clinical changes, identifying unexpressed patient needs, and ensuring continuity of care over time. This expertise emerged as a key element supporting the effective functioning of infusion units, contributing to the maintenance of care quality even in contexts characterized by organizational variability. In this setting, professional competence appears to complement existing organizational models, facilitating care delivery and enabling adaptation to diverse operational demands.

#### 3.2.4. Chair Occupancy as a System Bottleneck

A second construct concerned the recognition of chair time as the main bottleneck of infusion care. Participants emphasized that the critical issue was not only the pharmacological infusion time itself, but the total pathway time during which a chair remains occupied. This included pre-infusion assessment, premedication, latency, drug administration, monitoring, documentation, and turnover activities. From this perspective, chair occupancy was interpreted not simply as a logistical variable, but as the central limiting factor affecting capacity, scheduling flexibility, and throughput. Participants repeatedly linked prolonged pathways to saturation of available slots, concentration of activity in the morning hours, and the inability to respond more flexibly to patient needs.

#### 3.2.5. Separation Between Technical Time and Relational Time

A third major construct was the distinction between technical time and relational time. The Board clearly highlighted that the value of infusion care cannot be reduced to administration time alone. Participants distinguished between the time needed for technical and safety-related procedures and the time dedicated to clinical listening, education, therapeutic communication, and identification of patient-reported concerns. In traditional infusion models, these two dimensions often coexist within the same care episode. However, shorter administration pathways were seen as potentially disrupting this implicit overlap. For this reason, participants argued that the introduction of shorter infusions should not be interpreted as a reduction in care quality, but rather as a need to intentionally redesign how and when relational care is delivered. The main risk identified was not shorter time per se, but the absence of protected and structured opportunities for meaningful nurse–patient interaction.

#### 3.2.6. Shorter Infusion Time as an Enabler of Organizational Redesign

A fourth construct concerned the interpretation of shorter infusion therapies, particularly one-hour intravenous administration, as an organizational enabler rather than merely a time-saving option. Participants consistently acknowledged the potential of shorter pathways to improve chair turnover, release capacity, and facilitate more flexible scheduling, including the possibility of using previously underutilized time slots. At the same time, the Board stressed that these benefits would not be fully realized without broader process redesign. Shorter infusion time alone was not seen as sufficient to solve structural inefficiencies such as staff shortages, pharmacy delays, rigid scheduling models, or limited KPI tracking. Instead, it was interpreted as a high-leverage opportunity that could support transformation if combined with workflow reorganization, clearer separation of care phases, and greater nursing empowerment in patient assessment and pathway management.

#### 3.2.7. Hidden Organizational Burdens Beyond Measurable Workload

An additional cross-cutting construct concerned the existence of hidden burdens that are not always captured by quantitative metrics. Participants referred to shared spaces, interruptions, heterogeneous therapeutic protocols, patient-to-patient comparisons, lack of privacy, and fragmented communication flows as factors that increase complexity without necessarily appearing in standard workload indicators. These elements were perceived as amplifiers of cognitive load and as barriers to both efficiency and relational quality. In this sense, the qualitative discussion suggested that some of the most relevant determinants of infusion center performance are embedded in the organization of care environments and interactions, rather than in measurable task duration alone.

## 4. Discussion

The present study aimed to explore the organization of infusion centers for multiple sclerosis, with a particular focus on nursing workload, operational efficiency, and the perceived impact of infusion duration. Overall, the findings provide a comprehensive real-world overview of infusion care across Italy and highlight significant variability in organizational structures, resource availability, and workload distribution, as well as the presence of relevant inefficiencies within current infusion pathways. These results were further enriched by insights from the expert discussion, which confirmed that infusion centers function as complex adaptive systems where organizational constraints, professional expertise, and treatment characteristics continuously interact to shape both efficiency and quality of care [21].

### 4.1. Infusion Center Workload and Organizational Constraints

From a descriptive perspective, infusion centers were characterized by high patient volumes, limited staffing, and a substantial degree of multitasking. Nurses reported managing multiple patients simultaneously and supervising a considerable number of infusions per day, often within settings where resources and dedicated spaces were not uniformly available. These findings are consistent with previous studies showing that infusion services frequently operate under conditions of organizational strain, where increasing therapeutic demand is not matched by proportional resource expansion [22,23]. The high prevalence of administrative activities suggests that a substantial portion of nursing time is dedicated to non-clinical tasks, which may contribute to some degree of workflow fragmentation and reduced efficiency [21]. Observations from the Expert Nursing Board indicate that this pressure is often managed through the high level of nursing expertise, with experienced professionals supporting care delivery through adaptability and tacit knowledge, alongside existing organizational processes [24]. In this context, reducing infusion may represent one potential organizational strategy that could contribute to improving patient flow and freeing operational capacity, although this hypothesis requires confirmation in prospective comparative studies. Shorter infusion protocols could potentially reduce chair occupancy and overall treatment duration and may facilitate a more efficient allocation of available resources, as perceived by participating nurses [25]. Evidence from other infusion-based settings suggests that reductions in administration time can enhance throughput and optimize workflow efficiency, particularly when integrated within broader organizational improvements [26]. Importantly, in high-demand environments such as MS infusion centers, even modest reductions in infusion duration may have the potential to translate into gains in capacity, although this was not directly evaluated in the present study, enabling the treatment of additional patients without requiring proportional increases in staffing or infrastructure. Consequently, therapies with shorter infusion times could potentially support system capacity and facilitate a redistribution of nursing activities, if integrated within appropriate organizational models [27]. From the qualitative perspective, these potential gains were consistently interpreted not merely as time savings, but as opportunities to release organizational capacity and rebalance workload distribution across the day [28].

### 4.2. Complexity of Infusion Care and Workflow Inefficiencies

The analysis of the infusion process revealed that nursing care is distributed across multiple phases, with post-infusion monitoring representing the most time-consuming component [29,30]. This aligns with prior evidence indicating that infusion management is not only time-intensive but also cognitively demanding. Nurses are required to ensure precise drug administration and continuous monitoring for safety, often under conditions of high responsibility and stress [27]. As highlighted in previous literature, infusion-related care is shaped by a complex interaction between human and system factors, reinforcing the idea that it should not be interpreted solely as a time-based activity but rather as a multifaceted clinical process requiring sustained attention and coordination. Consistently, observations from the expert nursing board emphasized that infusion care cannot be reduced to a sequence of technical tasks but rather represents an integrated process in which clinical judgment, relational skills, and system constraints coexist and interact [31]. Importantly, descriptive findings also highlighted the presence of operational inefficiencies, including waiting times before infusion initiation and idle time between patients. Similar inefficiencies have been reported in other infusion-based settings, where delays and suboptimal scheduling reduce the effective utilization of available resources [32]. These aspects suggest that current infusion workflows are not fully optimized and that a portion of care time is lost due to organizational constraints rather than clinical necessity. Participants in the expert panel discussion further highlighted that these inefficiencies are often amplified by systemic factors such as shared spaces, interruptions, and heterogeneous care pathways, which increase complexity without being directly captured by traditional performance indicators [33]. In this scenario, therapies with shorter infusion times may help mitigate some of these inefficiencies, according to nurses’ perceptions, and potentially may contribute to reducing bottlenecks within the patient pathway and facilitating smoother scheduling and turnover of infusion chairs. Importantly, shorter pathways were perceived as particularly valuable when enabling a more flexible use of time slots, including previously underutilized periods such as midday or afternoon sessions [34].

### 4.3. Infusion Efficiency as a Function of Organizational Capacity Rather than Task Duration

Building on these observations, inferential analyses provided further insight into the determinants of infusion capacity. The results clearly indicate that infusion activity is primarily driven by structural and organizational factors rather than by the time required for individual nursing tasks [35]. Specifically, Operational Workload and Resource Availability were strongly associated with the number of infusions performed per day, while Time of Nursing Assistance was not. These findings are in line with previous research showing that healthcare system performance is largely influenced by macro-level organizational variables, such as staffing, infrastructure, and process organization, rather than by the duration of individual clinical activities [36]. This interpretation was strongly supported by insights from the expert discussion, which consistently framed infusion efficiency as a system-level property emerging from the interaction between resources, workflows, and patient flow management [37]. Non-parametric analyses further supported this interpretation, demonstrating that higher-volume centers were characterized by greater workload intensity and increased resource availability [38]. This suggests that infusion efficiency is closely linked to the ability of centers to scale resources and adapt organizational structures to patient demand. Conversely, centers with lower patient volumes showed reduced workload and resource levels, indicating a strong interdependence between activity volume and organizational capacity. The observed differences between centers appear to be primarily related to time management within infusion workflows rather than to variations in workload intensity or resource availability. In this context, therapies with shorter infusion durations, such as ublituximab, could represent a potential organizational opportunity for more streamlined care processes; however, this inference should be interpreted as a hypothesis rather than as evidence generated by the present study. This may be particularly relevant in settings without dedicated infusion infrastructure, where longer administration times can affect nursing assistance duration and daily workflow organization. Shorter infusion protocols could therefore enhance scheduling flexibility and improve the allocation of nursing time without altering overall workload levels. The comparison between centers with different activity volumes highlighted that nursing assistance time remains relatively stable, while operational workload and resource availability increase. In this context, reducing infusion time to approximately one hour, as with ublituximab, may represent a promising organizational hypothesis that could facilitate workflow optimization and scheduling flexibility. However, the present study did not directly compare infusion protocols, and these potential benefits require confirmation in prospective comparative studies. Observations from the expert nursing board further reinforced this relationship by highlighting how higher-volume centers tend to develop more adaptive organizational strategies, whereas lower-volume settings often rely more heavily on individual professional flexibility [39]. Interestingly, although the majority of nurses reported that prolonged infusion times had at least a moderate impact on workload, this perception was not associated with objective differences in workload, resource availability, or time of assistance. This discrepancy highlights a well-documented phenomenon in healthcare settings, where perceived workload is influenced not only by measurable task duration but also by organizational rigidity, time pressure, and workflow constraints [27]. In this context, longer infusion times may contribute to a sense of saturation and reduced flexibility, even if they do not directly increase the measurable time spent on nursing tasks. According to the expert board, this perception was interpreted as a consequence of limited flexibility and constrained scheduling capacity, rather than of increased task duration per se [40]. Taken together, these findings provide a nuanced understanding of the potential role of therapies with shorter infusion times. Our findings suggest that shorter infusion protocols could potentially act at the organizational level by facilitating patient flow and reducing bottlenecks, although this interpretation is based on nurses’ perceptions rather than objective comparative evidence. This interpretation is consistent with evidence from other clinical settings, where reducing treatment duration alone does not automatically decrease workload but can significantly improve system performance when combined with optimized workflows [32]. Importantly, insights from the expert discussion highlighted that shorter infusion times are most valuable when interpreted as enablers of pathway redesign, rather than as isolated efficiency gains [27]. In this perspective, therapies characterized by one-hour administration, such as ublituximab, may represent a potential opportunity to simplify some aspects of infusion pathway organization. Nevertheless, these considerations should be regarded as hypotheses derived from nurses’ perceptions and require validation through prospective comparative studies. By shortening patient occupancy time, these treatments could potentially allow a more flexible redistribution of nursing activities. Whether they reduce cognitive workload or improve care processes remains to be demonstrated in future comparative studies. Furthermore, by decreasing time constraints within the infusion pathway, shorter protocols may enhance the capacity of nurses to manage competing demands more effectively, ultimately improving both workflow sustainability and patient safety. At the same time, observations from the expert nursing board underscored the importance of explicitly separating technical time from relational time, ensuring that efficiency gains do not inadvertently reduce opportunities for patient interaction, assessment, and education. Importantly, if confirmed, such organizational improvements could translate into indirect benefits for nursing practice, including greater availability of time for patient assessment, education, and therapeutic communication, which are key components of high-quality, patient-centered care [11].

### 4.4. Strengths and Limitations

This study has several strengths that enhance the relevance and validity of its findings. First, it provides a comprehensive real-world overview of infusion center organization in multiple sclerosis across a national context, including a wide geographic distribution of centers. The high response rate (87.2%) supports the representativeness of the sample and strengthens the reliability of the results. Additionally, the use of an AI-assisted platform for data collection, qualitative comment analysis, expert board proceedings analysis, and report generation enhanced methodological consistency, ensured full traceability of the analytical process, and reduced potential biases associated with manual data processing and qualitative interpretation, while maintaining scientific oversight through systematic human validation of all AI-generated outputs. Second, the study integrates both descriptive and inferential analyses, allowing for a multidimensional understanding of infusion care that combines structural, operational, and perceptual aspects. The development of composite domains capturing key dimensions such as Time of Nursing Assistance, Operational Workload, and Resource Availability represents an additional strength, as it enables a more structured and interpretable evaluation of complex organizational dynamics. However, several limitations should be acknowledged. The cross-sectional design limits the ability to infer causal relationships between organizational factors and infusion capacity. All data were self-reported by nurses, which may introduce recall bias or subjective interpretation, particularly for time estimates and perceived workload. Although the sample is geographically diverse, it is restricted to Italian centers, which may limit the generalizability of findings to other healthcare systems with different organizational models. In addition, the composite indices used in the analysis, while conceptually grounded, were not externally validated and may not fully capture all dimensions of workload and resource availability. Furthermore, although the questionnaire underwent face and content validity assessment and pilot testing, it did not undergo a formal psychometric validation process. Therefore, it should be considered an exploratory organizational survey rather than a validated measurement instrument, and the findings should be interpreted within this context. Finally, the study did not directly measure patient-level outcomes or objective workflow metrics, such as actual chair turnover or time-motion data, which could further strengthen the interpretation of organizational efficiency.

## 5. Conclusions

In conclusion, this study provides a detailed real-world characterization of infusion center organization in multiple sclerosis, highlighting substantial variability in resources, workload, and operational efficiency across centers. The findings demonstrate that infusion capacity is primarily driven by structural and organizational factors, such as staffing levels and resource availability, rather than by the duration of individual nursing activities. Importantly, the results suggest that inefficiencies within infusion pathways—such as waiting times, idle chair periods, and administrative burden—play a significant role in limiting system performance. In this context, therapies with one-hour infusion protocols, such as ublituximab, may represent a potential opportunity to improve organizational flexibility and patient flow. However, because the present study explored nurses’ perceptions rather than directly comparing infusion protocols, these possible organizational advantages should be regarded as hypotheses that warrant confirmation in future prospective comparative studies. If confirmed in future studies, shorter infusion protocols could contribute to improving organizational flexibility and efficiency, potentially allowing more time for clinical assessment, education, and therapeutic communication. This includes increased availability of time for clinical assessment, education, and therapeutic communication, which are essential components of high-quality care in multiple sclerosis. The integration of AI-assisted analytical tools within a structured expert consultation framework demonstrated the feasibility and value of combining advanced digital methodologies with clinical expertise to generate robust, reproducible, and actionable evidence for healthcare decision-making. Future research should focus on integrating organizational interventions with innovative therapeutic strategies, as well as on evaluating the impact of reduced infusion times using objective workflow measures and patient-reported outcomes, to further inform the development of efficient and patient-centered infusion care models.

## Figures and Tables

**Table 1:** Participant Characteristics and Clinical Activity N = 68.

Variable	Mean ± SD	
Years of experience as nurse	21.54 ± 10.52	
Years of experience in MS	8.66 ± 7.97	
Infusions per day	7.79 ± 5.36	
Area	Regions Included	N (%)
Northern Italy	Lombardia, Veneto, Emilia-Romagna, Piemonte, Liguria, Friuli-Venezia Giulia	19 (27.9%)
Central Italy	Toscana, Lazio, Marche, Umbria	24 (35.3%)
Southern Italy and Islands	Puglia, Campania, Sicilia, Sardegna, Calabria, Basilicata	25 (36.8%)

Geographic distribution is reported according to macro-areas of Italy.

**Table 2:** Organizational Structure and Resource Availability.

Variable	Mean ± SD
MS patients treated per month	95.82 ± 87.81
Infusion chairs	5.96 ± 3.42
Active infusion stations	7.06 ± 7.24
Infusion devices (pumps)	5.87 ± 4.78
Nurses per infusion shift	2.44 ± 1.39
Patients manageable per nurse (per shift)	5.98 ± 4.91
Patients managed simultaneously	2.54 ± 3.73
Patients supervised Infusions per day	7.79 ± 5.36

Organizational characteristics of infusion centers, including infrastructure, staffing levels, and patient management capacity.

**Table 3:** Organizational Characteristics (Categorical Variables).

Variable	Category	N (%)
Use of infusion pumps	Yes	59 (86.8%)
	Partially	8 (11.8%)
	No	1 (1.5%)
Access to standardized protocols	Yes	66 (97.1%)
	No	2 (2.9%)
Dedicated infusion area	Yes	39 (57.4%)
	No	29 (42.6%)
Monthly patient volume	>50	26 (38.2%)
	30–50	19 (27.9%)
	10–30	19 (27.9%)
	<10	4 (5.9%)
Nursing shifts	Fixed	51 (75.0%)
	Flexible	10 (14.7%)
	Variable	7 (10.3%)
Administrative activities between infusions	Yes	60 (88.2%)
	No	8 (11.8%)

Distribution of key organizational and structural characteristics across participating centers.

**Table 4:** Infusion Process and Time Allocation (minutes).

Variable	Mean ± SD
Preparation time	13.90 ± 6.87
Patient reception and assessment	16.31 ± 9.52
Vital signs, premedication, and setup	27.74 ± 21.15
Post-infusion monitoring	52.50 ± 21.54
Post-infusion management	17.37 ± 12.30
Patient education and relationship	20.66 ± 13.71
Caregiver communication	14.03 ± 8.36
Bureaucratic activities	18.07 ± 17.19
Waiting time before infusion	20.85 ± 14.61
Chair idle time between infusions	19.03 ± 22.38

Time required for each phase of the infusion process, expressed in minutes. These variables contribute to the estimation of overall nursing time and operational efficiency.

**Table 5:** Perceived Impact and Access to Infusion Care.

Variable	Category	N (%)
Impact of long infusion times on workload	Moderate	35 (51.5%)
	High	17 (25.0%)
	Low	9 (13.2%)
	No Impact	7 (10.3%)
Delay in access to first infusion	Occasional	36 (52.9%)
	Frequent	11 (16.2%)
	None	21 (30.9%)
Delay in subsequent infusions	None	45 (66.2%)
	Occasional	22 (32.4%)
	Frequent	1 (1.5%)
Delay on scheduled infusion day	Occasional	29 (42.6%)
	None	35 (51.5%)
	Frequent	4 (5.9%)

Nurses’ perceptions of the impact of infusion duration on workload and reported delays in access to infusion therapies.

**Table 6:** Derived Domain.

Domain	Mean ± SD
Time of Nursing Assistance (minutes)	22.11 ± 6.91
Operational Workload (composite index)	5.71 ± 4.23
Resource Availability (composite index)	5.15 ± 3.14

Time of Nursing Assistance (minutes) represents the mean time spent across key infusion-related activities, including preparation of the infusion setting, patient reception and assessment, vital signs monitoring and premedication, post-infusion monitoring, post-infusion management, and patient education and relationships. Operational Workload (composite index) reflects the nursing workload intensity and was calculated based on the average number of patients manageable per nurse per shift, the number of patients managed simultaneously. Resource Availability (composite index) reflects the structural and staffing capacity of infusion centers and was calculated based on the number of active infusion stations, infusion devices (pumps), and nurses per infusion shift.

**Table 7:** Multiple Linear Regression Analysis Predicting Number of Infusions per Day (N = 68).

Predictor	B (Unstandardized)	SE	β (Standardized)	t	*p*	95% CI
**Intercept**	6.17	2.45	—	2.52	0.014	—
Time of Nursing Assistance	0.03	0.08	0.04	0.39	0.697	−0.162
Operational Workload	0.01	0.13	0.00	0.04	0.965	−0.197
Resource Availability	0.79	0.18	0.46	4.41	<0.001	0.253
**Monthly Patient Volume (ref: 30–50 patients/month)**						
>50 vs. 30–50	−0.51	1.33	−0.09	−0.38	0.704	−0.592
10–30 vs. 30–50	−3.54	1.41	−0.66	−2.51	0.015	−1.185
<10 vs. 30–50	−5.33	2.38	−0.99	−2.24	0.029	−1.885
**Perceived Impact of Long Infusion Times on Nursing Burden (ref: High impact)**						
Moderate vs. High	−1.60	1.29	−0.30	−1.24	0.218	−0.780
Low vs. High	−3.91	1.80	−0.73	−2.17	0.034	−1.400
No Impact vs. High	−3.04	1.91	−0.57	−1.59	0.117	−1.281
Model	R	R^2^	Adjusted R^2^			
Model 1	0.683	0.467	0.384			

Abbreviations: B = unstandardized regression coefficient; SE = standard error; β = standardized regression coefficient; CI = confidence interval.

**Table 8:** Differences in Time of Nursing Assistance, Operational Workload, and Resource Availability According to Monthly Patient Volume.

Variable	χ^2^	df	*p*	ε^2^	Significant Pairwise Comparisons (Bonferroni-Adjusted)
Time of Nursing Assistance	3.61	3	0.306	0.054	No significant differences between groups
Operational Workload	11.55	3	0.009	0.172	<10 vs. 30–50 patients/month (*p* = 0.033); <10 vs. >50 patients/month (*p* = 0.019)
Resource Availability	11.20	3	0.011	0.167	10–30 vs. 30–50 patients/month (*p* = 0.024); 10–30 vs. >50 patients/month (*p* = 0.020)

Abbreviations: df = degrees of freedom; ε^2^ = epsilon-squared effect size.

**Table 9:** Comparison between centers with and without a dedicated infusion area.

Variable	No Impact (n = 29) Mean ± SD	High/Moderate Impact (n = 39) Mean ± SD	Mann–Whitney U	*p*
Time of Nursing Assistance	24.34 ± 7.36	20.46 ± 6.12	399	0.038
Operational Workload	5.59 ± 3.65	5.79 ± 4.65	556	0.910
Resource Availability	5.48 ± 4.17	4.90 ± 2.10	534	0.697

Values are expressed as mean ± standard deviation. Group comparisons were performed using the Kruskal–Wallis test. A *p*-value < 0.05 was considered statistically significant.

## Data Availability

The data presented in this study are available on reasonable request from the corresponding author. The data are not publicly available due to privacy and ethical restrictions.

## Supplementary Materials

The following supporting information can be downloaded at: https://www.mdpi.com/article/10.3390/healthcare14142094/s1, File S1: Questionnaire on Infusion Center Organization, Nursing Workload, and Perceived Impact of Reduced Infusion Time in Multiple Sclerosis.

## Abbreviations

The following abbreviations are used in this manuscript:

AI	Artificial Intelligence
AIFA	Italian Medicines Agency
AISM	Italian Multiple Sclerosis Association
APC	Article Processing Charge
CRSwNP	Chronic Rhinosinusitis with Nasal Polyps
GDPR	General Data Protection Regulation
KPI	Key Performance Indicator
MS	Multiple Sclerosis
MSNICE	Multiple Sclerosis Nursing to Improve Care and Education
SD	Standard Deviation
SE	Standard Error

## Appendix A

Questionnaire on Infusion Center Organization, Nursing Workload, and Perceived Impact of Reduced Infusion Time in Multiple Sclerosis

Section A. Operator Profile

1. Years of experience as a nurse

Response: Numeric field

2. Years of specific experience in multiple sclerosis

Response: Numeric field

3. Average number of infusions supervised per day

Response: Numeric field

4. Do you use infusion pumps for infusion management?

Response options:

- Yes
- No
- Partially

5. Do you have access to standardized protocols for infusion care management?

Response options:

- Yes
- No

Section B. General Information About the Center

6. Name of the center

Response: Open text

7. Region

Response options:

- Abruzzo
- Basilicata
- Calabria
- Campania
- Emilia-Romagna
- Friuli-Venezia Giulia
- Lazio
- Liguria
- Lombardia
- Marche
- Molise
- Piemonte
- Puglia
- Sardegna
- Sicilia
- Toscana
- Trentino-Alto Adige
- Umbria
- Valle d’Aosta
- Veneto

8. Does your center have an infusion area dedicated exclusively to patients with MS?

Response options:

- Yes
- No

9. Does your center have an infusion area shared with other departments or with treatments for other neurological diseases?

Response options:

- Yes
- No

10. On average, how many patients with MS are treated with infusion drugs each month in your center?

Response: Numeric field

11. How many infusion chairs are available in your Day Hospital?

Response: Numeric field

12. How many infusion stations can be active simultaneously?

Response: Numeric field

13. Number of automated infusion devices available (infusion pumps)

Response: Numeric field

14. On which days and at what times is the infusion service active?

Response matrix by day and time slot:

- Monday morning
- Monday afternoon
- Tuesday morning
- Tuesday afternoon
- Wednesday morning
- Wednesday afternoon
- Thursday morning
- Thursday afternoon
- Friday morning
- Friday afternoon
- Saturday morning
- Saturday afternoon
- Sunday morning
- Sunday afternoon

For each slot:

- Open
- Closed

15. Average monthly volume of patients treated with monoclonal antibodies

Response options:

- Fewer than 10
- Between 10 and 30
- Between 30 and 50
- More than 50

16. According to the SmPC, for infusions after the first one, what is the average infusion time in minutes for rituximab?

Response: Numeric field

17. According to the SmPC, for infusions after the first one, what is the average infusion time in minutes for ocrelizumab?

Response: Numeric field

18. According to the SmPC, for infusions after the first one, what is the average infusion time in minutes for ublituximab?

Response: Numeric field

Section C. Resources and Operational Times

19. Average number of nurses present per infusion shift

Response: Numeric field

20. Estimated number of hours per week you stay beyond scheduled working hours because of infusions

Response: Numeric field

21. Nursing shifts are:

Response options:

- Fixed
- Flexible on a weekly basis
- Variable according to workload

22. Average number of patients manageable by one nurse per shift, based on your experience

Response: Numeric field

23. How many minutes are needed to prepare the chair and materials for infusion?

Response: Numeric field

24. How many minutes are needed for patient reception and initial assessment?

Response: Numeric field

25. How many minutes are needed for assessment of vital signs, premedication, and infusion set preparation?

Response: Numeric field

26. How many minutes are needed to manage post infusion monitoring activities during first infusions according to protocol?

Response: Numeric field

27. How many minutes are needed to manage post infusion activities, such as discharge preparation and booking the next appointment?

Response: Numeric field

28. Estimated minutes dedicated to patient education and relationship, before and or after infusion

Response: Numeric field

29. Estimated minutes dedicated to communication with the caregiver, if present

Response: Numeric field

30. Are there bureaucratic or administrative activities to be completed between one infusion and the next?

Response options:

- Yes
- No

31. If yes, how many minutes do these activities usually require?

Response: Numeric field

32. How many patients can one nurse manage in parallel during an infusion shift?

Response: Numeric field

33. How intense is the perceived workload during infusions?

Response: 1 to 5 scale

- 1 = Very light workload
- 2 = Light workload
- 3 = Moderate workload
- 4 = High workload
- 5 = Very high workload

34. During which time slot are infusions mostly concentrated?

Response options:

- Morning, 08:00 to 10:00
- Morning, 10:00 to 12:00
- Morning, 12:00 to 14:00
- Afternoon, 14:00 to 16:00
- Afternoon, 16:00 to 18:00

35. What is the average frequency of no shows, meaning patients who do not attend?

Response options:

- None
- 1 to 5%
- 5 to 10%
- More than 10%

36. Estimated percentage of patients who are employed and have specific scheduling needs

Response: Numeric field, percentage

37. How are infusion appointments scheduled?

Response options:

- Fixed appointment times
- Open access within defined hours, for example from 08:00 to 14:00
- Other, please specify in comments

38. To what extent do drugs with infusion protocols lasting more than one hour affect nursing activities and the capacity to treat patients?

Response options:

- Not at all
- A little
- Moderately
- A lot
- Completely

Section D. Infusion Reactions and Infusion Protocol Management

39. How often do infusion related reactions occur during the first infusion?

Response options:

- Never
- Occasionally, fewer than 10
- Frequently, more than 10

40. How often do infusion related reactions occur in infusions after the first one?

Response options:

- Never
- Occasionally, fewer than 10
- Frequently, more than 10

41. Please estimate the percentage of patients treated with ocrelizumab who receive the short infusion protocol, 2 h, compared with the standard protocol, 3.5 h

Response: Numeric field, percentage

Section E. Key Performance Indicators and Management Indicators

42. On average, how many patients can be treated on a single chair in one day?

Response: Numeric field

43. Are there significant waiting times for patients to access their first infusion, that is, time between prescription and first administration?

Response options:

- No
- Yes, occasional
- Yes, frequent
- Yes, critical

44. Are there significant waiting times for patients to access subsequent infusions?

Response options:

- No
- Yes, occasional
- Yes, frequent
- Yes, critical

45. Are there significant waiting times for patients to access infusion on the scheduled day?

Response options:

- No
- Yes, occasional
- Yes, frequent
- Yes, critical

46. Estimate the waiting time in minutes from patient check in to infusion start

Response: Numeric field

47. How many days of waiting are there, on average, between prescription and actual infusion?

Response: Numeric field

48. On average, how long does a chair remain unused between two infusions, operational gap in minutes?

Response: Numeric field

49. Chair turnover rate indicates how many infusions or patients are managed on each chair over a given period of time, such as per day or per week. It is an indicator of efficiency in the use of infusion stations. How is it assessed in your center?

Response options:

- Regularly calculated
- Estimated based on experience
- Not assessed

50. If a drug with an infusion time reduced to approximately one hour, for example ublituximab, were introduced, what would be the perceived impact in the following areas?

Response matrix, score from 1 to 5:

Rows:

- Capacity to care for a greater number of patients
- Enhancement of nursing activity, such as patient relationship, needs assessment, and similar aspects
- Organization and efficiency of the infusion room, such as shifts, spaces, and patient flow

Columns:

- 1 = Negative impact or worsening
- 2 = Slightly negative impact
- 3 = No impact
- 4 = Positive impact
- 5 = Very positive impact

Section F. Qualitative and Critical Aspects

51. What are the main bottlenecks in infusion management?

Response options, multiple choice if needed:

- Staff shortage
- Limited space
- Overlap with other therapies or treatments
- Drug preparation times
- Long infusion times
- Other, please specify in comments

52. Please describe the main difficulties in managing current infusions

Response: Open text

53. If infusion time were reduced to one hour, what impact would you expect on the center’s daily capacity?

Response: Numeric field

54. If infusion time were reduced to one hour, what impact would you expect on staff shifts?

Response options:

- More flexible
- Unchanged
- More stressful

55. If infusion time were reduced to one hour, what impact would you expect on improvement in quality of care?

Response: 1 to 5 scale

56. If infusion time were reduced to one hour, what impact would you expect on time available for other nursing tasks, in terms of minutes gained per shift?

Response: Numeric field

57. Which KPIs do you consider most relevant to assess the efficiency of your unit?

Response: Open text

Section G. Acceptance and Simulation

58. Please indicate what you would find most useful to assess through a software system able to:

Response matrix, score from 1 to 5

Rows:

- Simulate the effect of reduced infusion time in facilitating nursing work
- Optimize chair turnover
- Estimate the impact on nursing workload and staffing
- Support managerial decisions with dynamic scenarios in relation to patient satisfaction

Columns:

- 1 = Not useful at all
- 2 = Slightly useful
- 3 = Moderately useful
- 4 = Useful
- 5 = Very useful

59. Would you support the introduction of an innovative model for infusion room management in light of new drugs with reduced infusion times, if clinically equivalent?

Response options:

- Yes
- No
- It depends

60. What impact would you expect on the nursing team if infusion time were reduced to one hour?

Response: Open text
