# Supplementary material for: Optimizing Infusion Center Efficiency in Multiple Sclerosis: A Nursing Multicenter Survey on Organizational Impact of Ublituximab
Source: Healthcare (Basel). 2026 Jul 13;14(14):2094. doi: 10.3390/healthcare14142094 (PMC13411801; doi:10.3390/healthcare14142094)
Supplement: Supplementary file 1 [file healthcare-14-02094-s001.zip › healthcare-4329181-supplementary.pdf]

Supplementary file S1: Questionnaire on Infusion Center Organization, Nursing Workload, and Perceived Impact of Reduced Infusion Time in Multiple Sclerosis

Section A. Operator Profile

1. Years of experience as a nurse

Response: Numeric field

2. Years of specific experience in multiple sclerosis

Response: Numeric field

3. Average number of infusions supervised per day

Response: Numeric field

4. Do you use infusion pumps for infusion management?

Response options:

Yes

No

Partially

5. Do you have access to standardized protocols for infusion care management?

Response options:

Yes

No

Section B. General Information About the Center

6. Name of the center

Response: Open text

7. Region

Response options:

Abruzzo

Basilicata

Calabria

Campania

Emilia-Romagna

Friuli-Venezia Giulia

Lazio

Liguria

Lombardia

Marche

Molise

Piemonte

Puglia

Sardegna

Sicilia

Toscana

Trentino-Alto Adige

Umbria

Valle d'Aosta

Veneto

8. Does your center have an infusion area dedicated exclusively to patients with MS?

Response options:

Yes

No

9. Does your center have an infusion area shared with other departments or with treatments for other neurological diseases?

Response options:

Yes

No

10. On average, how many patients with MS are treated with infusion drugs each month in your center?

Response: Numeric field

11. How many infusion chairs are available in your Day Hospital?

Response: Numeric field

12. How many infusion stations can be active simultaneously?

Response: Numeric field

13. Number of automated infusion devices available (infusion pumps)

Response: Numeric field

14. On which days and at what times is the infusion service active?

Response matrix by day and time slot:

Monday morning

Monday afternoon

Tuesday morning

Tuesday afternoon

Wednesday morning

Wednesday afternoon

Thursday morning

Thursday afternoon

Friday morning

Friday afternoon

Saturday morning

Saturday afternoon

Sunday morning

Sunday afternoon

For each slot:

Open

Closed

15. Average monthly volume of patients treated with monoclonal antibodies

Response options:

Fewer than 10

Between 10 and 30

Between 30 and 50

More than 50

16. According to the SmPC, for infusions after the first one, what is the average infusion time in minutes for rituximab?

Response: Numeric field

17. According to the SmPC, for infusions after the first one, what is the average infusion time in minutes for ocrelizumab?

Response: Numeric field

18. According to the SmPC, for infusions after the first one, what is the average infusion time in minutes for ublituximab?

Response: Numeric field

Section C. Resources and Operational Times

19. Average number of nurses present per infusion shift

Response: Numeric field

20. Estimated number of hours per week you stay beyond scheduled working hours because of infusions

Response: Numeric field

21. Nursing shifts are:

Response options:

Fixed

Flexible on a weekly basis

Variable according to workload

22. Average number of patients manageable by one nurse per shift, based on your experience

Response: Numeric field

23. How many minutes are needed to prepare the chair and materials for infusion?

Response: Numeric field

24. How many minutes are needed for patient reception and initial assessment?

Response: Numeric field

25. How many minutes are needed for assessment of vital signs, premedication, and infusion set preparation?

Response: Numeric field

26. How many minutes are needed to manage post infusion monitoring activities during first infusions according to protocol?

Response: Numeric field

27. How many minutes are needed to manage post infusion activities, such as discharge preparation and booking the next appointment?

Response: Numeric field

28. Estimated minutes dedicated to patient education and relationship, before and or after infusion

Response: Numeric field

29. Estimated minutes dedicated to communication with the caregiver, if present

Response: Numeric field

30. Are there bureaucratic or administrative activities to be completed between one infusion and the next?

Response options:

Yes

No

31. If yes, how many minutes do these activities usually require?

Response: Numeric field

32. How many patients can one nurse manage in parallel during an infusion shift?

Response: Numeric field

33. How intense is the perceived workload during infusions?

Response: 1 to 5 scale

1 = Very light workload

2 = Light workload

3 = Moderate workload

4 = High workload

5 = Very high workload

34. During which time slot are infusions mostly concentrated?

Response options:

Morning, 08:00 to 10:00

Morning, 10:00 to 12:00

Morning, 12:00 to 14:00

Afternoon, 14:00 to 16:00

Afternoon, 16:00 to 18:00

35. What is the average frequency of no shows, meaning patients who do not attend?

Response options:

None

1 to 5%

5 to 10%

More than 10%

36. Estimated percentage of patients who are employed and have specific scheduling needs

Response: Numeric field, percentage

37. How are infusion appointments scheduled?

Response options:

Fixed appointment times

Open access within defined hours, for example from 08:00 to 14:00

Other, please specify in comments

38. To what extent do drugs with infusion protocols lasting more than one hour affect nursing activities and the capacity to treat patients?

Response options:

Not at all

A little

Moderately

A lot

Completely

Section D. Infusion Reactions and Infusion Protocol Management

39. How often do infusion related reactions occur during the first infusion?

Response options:

Never

Occasionally, fewer than 10

Frequently, more than 10

40. How often do infusion related reactions occur in infusions after the first one?

Response options:

Never

Occasionally, fewer than 10

Frequently, more than 10

41. Please estimate the percentage of patients treated with ocrelizumab who receive the short infusion protocol, 2 h, compared with the standard protocol, 3.5 h

Response: Numeric field, percentage

Section E. Key Performance Indicators and Management Indicators

42. On average, how many patients can be treated on a single chair in one day?

Response: Numeric field

43. Are there significant waiting times for patients to access their first infusion, that is, time between prescription and first administration?

Response options:

No

Yes, occasional

Yes, frequent

Yes, critical

44. Are there significant waiting times for patients to access subsequent infusions?

Response options:

No

Yes, occasional

Yes, frequent

Yes, critical

45. Are there significant waiting times for patients to access infusion on the scheduled day?

Response options:

No

Yes, occasional

Yes, frequent

Yes, critical

46. Estimate the waiting time in minutes from patient check in to infusion start

Response: Numeric field

47. How many days of waiting are there, on average, between prescription and actual infusion?

Response: Numeric field

48. On average, how long does a chair remain unused between two infusions, operational gap in minutes?

Response: Numeric field

49. Chair turnover rate indicates how many infusions or patients are managed on each chair over a given period of time, such as per day or per week. It is an indicator of efficiency in the use of infusion stations. How is it assessed in your center?

Response options:

Regularly calculated

Estimated based on experience

Not assessed

50. If a drug with an infusion time reduced to approximately one hour, for example ublituximab, were introduced, what would be the perceived impact in the following areas?

Response matrix, score from 1 to 5:

Rows:

Capacity to care for a greater number of patients

Enhancement of nursing activity, such as patient relationship, needs assessment, and similar aspects

Organization and efficiency of the infusion room, such as shifts, spaces, and patient flow

Columns:

1 = Negative impact or worsening

2 = Slightly negative impact

3 = No impact

4 = Positive impact

5 = Very positive impact

Section F. Qualitative and Critical Aspects

51. What are the main bottlenecks in infusion management?

Response options, multiple choice if needed:

Staff shortage

Limited space

Overlap with other therapies or treatments

Drug preparation times

Long infusion times

Other, please specify in comments

52. Please describe the main difficulties in managing current infusions

Response: Open text

53. If infusion time were reduced to one hour, what impact would you expect on the center's daily capacity?

Response: Numeric field

54. If infusion time were reduced to one hour, what impact would you expect on staff shifts?

Response options:

More flexible

Unchanged

More stressful

55. If infusion time were reduced to one hour, what impact would you expect on improvement in quality of care?

Response: 1 to 5 scale

56. If infusion time were reduced to one hour, what impact would you expect on time available for other nursing tasks, in terms of minutes gained per shift?

Response: Numeric field

57. Which KPIs do you consider most relevant to assess the efficiency of your unit?

Response: Open text

Section G. Acceptance and Simulation

58. Please indicate what you would find most useful to assess through a software system able to:

Response matrix, score from 1 to 5

Rows:

Simulate the effect of reduced infusion time in facilitating nursing work

Optimize chair turnover

Estimate the impact on nursing workload and staffing

Support managerial decisions with dynamic scenarios in relation to patient satisfaction

Columns:

1 = Not useful at all

2 = Slightly useful

3 = Moderately useful

4 = Useful

5 = Very useful

59. Would you support the introduction of an innovative model for infusion room management in light of new drugs with reduced infusion times, if clinically equivalent?

Response options:

Yes

No

It depends

60. What impact would you expect on the nursing team if infusion time were reduced to one hour?

Response: Open text
